# Deaths of Noted Physicians

**Published:** 1867-10

**Authors:** 


### *Deaths of Noted Physicians.*

Among French physicians—Gibert, Jobert, Trousseau, Follin, Velpeau, Rayer—all deceased within a single year. The death of Faraday alone, in England, makes the year one to be remembered with sorrow. In this country, death has been almost equally unsparing of names noted in the profession. On the whole, it has not been a year to be "marked by a white stone."

